# ERRATUM: Sertraline plus repetitive transcranial magnetic stimulation for the treatment of postpartum depression / Evaluation of cinitapride's efficacy and safety in treating functional dyspepsia with overlapping symptoms: a real-world study in Chinese healthcare settings / Conditions of vulnerability to the inadequate treatment of bronchiolitis

**DOI:** 10.1590/1806-9282.20241001-ER

**Published:** 2025-09-19

In the manuscript: Zhang Z, Luo T, Yu J. **Sertraline plus repetitive transcranial magnetic stimulation for the treatment of postpartum depression**. Rev Assoc Med Bras. 2025;71(4):e20241001. https://doi.org/10.1590/1806-9282.20241001

**Where it read:**

Zhengyu Zhang

**It should read:**

Zhenyu Zhang

In the manuscript: Yan B, Wang S, Chen J, Zhong X, Wang B, Wu Y, et al.. **Evaluation of cinitapride's efficacy and safety in treating functional dyspepsia with overlapping symptoms: a real-world study in Chinese healthcare settings**. Rev Assoc Med Bras. 2025;71(3):e20241628. https://doi.org/10.1590/1806-9282.20241628

**Where it read:**

Bing Yan^1,2†^, Shimin Wang^2†^, Jian Chen^3^, Xiaolin Zhong^4^, Bangmao Wang^5^,

Yumei Wu^6^, Ji Wu^7^, Nina Zhang^8^, Jing Sun^2^*, Duowu Zou^2^*

^1^Chongqing Medical University, The Second Affiliated Hospital, Department of Gastroenterology – Chongqing, China.

^2^Shanghai Jiao Tong University, School of Medicine, Ruijin Hospital, Department of Gastroenterology – Shanghai, China.

^3^Zhaoqing First People's Hospital, Department of Gastroenterology – Zhaoqing, China.

^4^Affiliated Hospital of Southwest Medical University, Department of Gastroenterology – Luzhou, China.

^5^Tianjin Medical University General Hospital, Department of Gastroenterology – Tianjin, China.

^6^The Third People's Hospital of Shenzhen, Department of Gastroenterology – Shenzhen, China.

^7^Foshan Fosun Chancheng Hospital, Department of Gastroenterology – Foshan, China.

^8^Nanjing Drum Tower Hospital (The Affiliated Hospital of Nanjing University Medical School), Department of Gastroenterology – Nanjing, China.

**It should read:**

Bing Yan^1,2†^, Shimin Wang^1†^, Jian Chen^3^, Xiaolin Zhong^4^, Bangmao Wang^5^,

Yumei Wu^6^, Ji Wu^7^, Nina Zhang^8^, Jing Sun^1^*, Duowu Zou^1^*

^1^Shanghai Jiao Tong University, School of Medicine, Ruijin Hospital, Department of Gastroenterology – Shanghai, China.

^2^Chongqing Medical University, The Second Affiliated Hospital, Department of Gastroenterology – Chongqing, China.

^3^Zhaoqing First People's Hospital, Department of Gastroenterology – Zhaoqing, China.

^4^Affiliated Hospital of Southwest Medical University, Department of Gastroenterology – Luzhou, China.

^5^Tianjin Medical University General Hospital, Department of Gastroenterology – Tianjin, China.

^6^The Third People's Hospital of Shenzhen, Department of Gastroenterology – Shenzhen, China.

^7^Foshan Fosun Chancheng Hospital, Department of Gastroenterology – Foshan, China.

^8^Nanjing Drum Tower Hospital (The Affiliated Hospital of Nanjing University Medical School), Department of Gastroenterology – Nanjing, China.

In the manuscript: Naves KC, Vieira SE. **Conditions of vulnerability to the inadequate treatment of bronchiolitis**. Rev Assoc Med Bras. 2020;66(2):187-193. https://doi.org/10.1590/1806-9282.66.2.187

**Where it read:**

Kattia Cristina Neves

**How to cite:** Neves KC, Vieira SE.

**It should read:**

Kattia Cristina Naves

**How to cite:** Naves KC, Vieira SE.

Roseli Nomura

José Maria Soares Júnior

*Scientifıc Editor*

